# Transcallosal, transchoroidal clipping of a hypothalamic collateral vessel aneurysm in Moyamoya disease

**DOI:** 10.1007/s00701-020-04335-4

**Published:** 2020-04-19

**Authors:** Wing Mann Ho, Alice Stephanie Görke, Florian Dazinger, Bettina Pfausler, Elke R. Gizewski, Ondra Petr, Claudius Thomé

**Affiliations:** 1 Department of Neurosurgery, Medical University Innsbruck, Anichstrasse 35, 6020 Innsbruck, Austria; 2 Department of Neuroradiology, Medical University Innsbruck, Anichstrasse 35, 6020 Innsbruck, Austria; 3 Department of Neurology, Medical University Innsbruck, Anichstrasse 35, 6020 Innsbruck, Austria

**Keywords:** Moyamoya, Aneurysm, Transcallosal approach, Clipping, Collateral vessel

## Abstract

Peripheral collateral vessel aneurysms in Moyamoya disease (MMD) remain difficult to treat due to their deep location, small size, and vascular fragility. We report the case of an aneurysm localized in the hypothalamus, which was rapidly increasing in size with repeated hemorrhage despite revascularization surgery. Aneurysm clipping was performed to prevent further progress and rerupture with favorable outcome. To our best knowledge, this is the first description of a hypothalamic aneurysm in MMD being clipped via a transcallosal, transchoroidal approach through the third ventricle.

**Electronic supplementary material:**

The online version of this article (10.1007/s00701-020-04335-4) contains supplementary material, which is available to authorized users.

## Introduction

Moyamoya-like disease (MMD) is characterized by stenotic or hypoplastic internal carotid arteries (ICA) leading to altered flow dynamics and increased wall shear stress. This pathophysiologic mechanism is proposed to induce the formation of a fragile collateral network of Moyamoya vessels and associated aneurysms [18]. Those collateral aneurysms are usually small in size (2–3 mm) but have been associated with intraventricular hemorrhage (IVH) [2, 13–15]. Surgical revascularization has been observed with following obliteration of collateral artery aneurysms. The treatment of deep-seated peripheral artery aneurysms is technically highly challenging, with only few cases reported with favorable outcomes [9].

To our best knowledge, this is the first description of a collateral vessel aneurysm localized in the hypothalamus being clipped via a transcallosal, transchoroidal approach.

## Case report

A 37 years-old female patient was admitted to the emergency room due to sudden severe headache with nausea, vomiting, and signs of meningeal irritation. She presented with anisocoria with a dilated right-sided pupil and a mild left-sided central facial palsy.

**Fig. 1:**
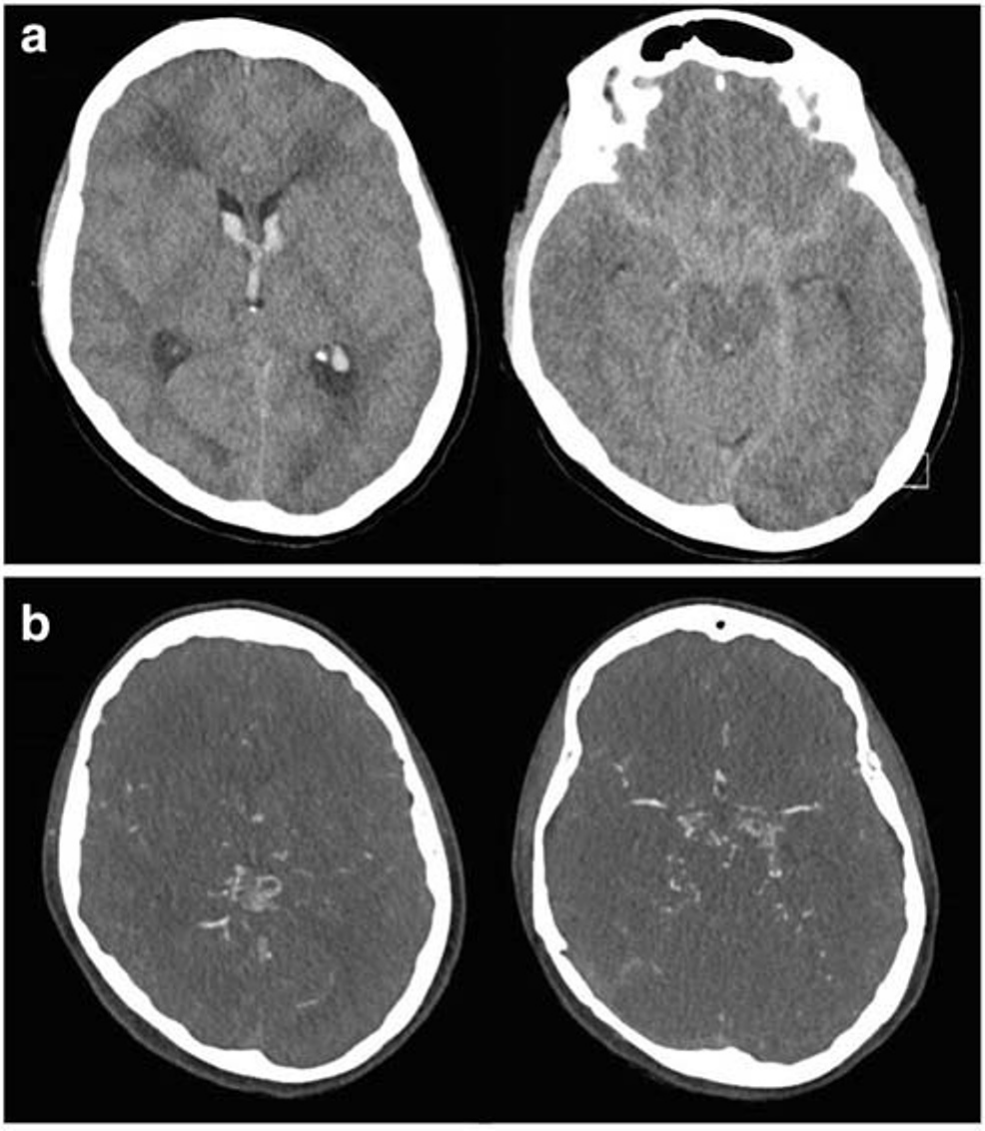
**a** The initial CT scan demonstrating ICH in the right hypothalamus with IVH and SAH in the basal cisterns, scored as Hunt and Hess 3/Fisher 4. Additionally, signs of diffuse leukoencephalopathy in both hemispheres with spotty calcification were observed. **b** The CT angiogram demonstrated stenosis of both distal internal carotid arteries (ICA) and a proximally dilated posterior communicating artery, which appeared occluded after the P1 segment. The middle (MCA) and anterior cerebral arteries (ACA) were partially fed by collaterals with a rete of collateral moyamoya vessels

The initial computed tomography (CT) showed intracerebral hemorrhage (ICH) in the right hypothalamus with intraventricular hemorrhage (IVH) and subarachnoid hemorrhage (SAH) in the basal cisterns with a Hunt and Hess score of 3 and Fisher score of 4 (Fig. 1a). Additionally, CT angiography (CTA) was conducted arousing suspicion of MMD with a network of dense collateral arterial vessels (Fig. 1b).

**Fig. 2:**
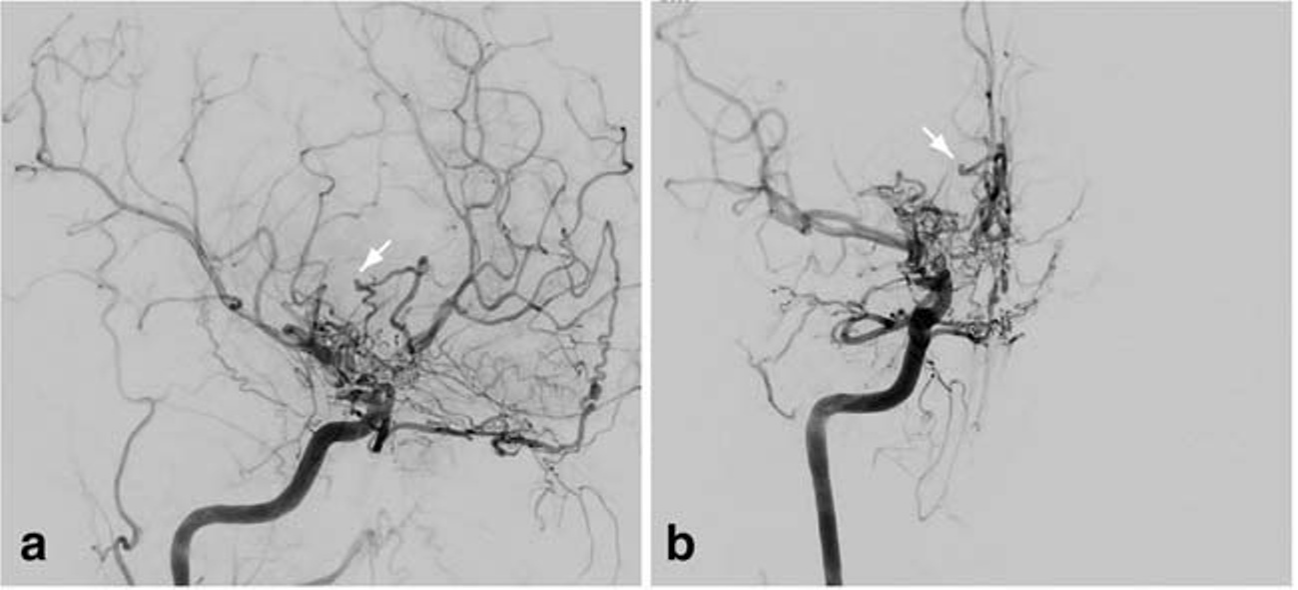
The initial DSA confirming the network of moyamoya-pattern collateral vessels. The arrow points to the aneurysm and suspected bleeding source. Severe stenosis of both ICA and the A1 segments and moderate stenosis of the left MCA were obvious with pronounced collaterals between the ICAs, both proximal ACAs and the right posterior communicating artery (PCOM). The dilated perforating vessels in the basal ganglia and hypothalamus were supposed to be caused by hypoperfusion due to MMD. One distinctive large collateral vessel was present between the reticular collaterals of the distal right-sided ICA and the ACA territory bearing a small aneurysm

The patient was admitted to the intensive care unit (ICU) and digital subtraction angiography (DSA) confirmed severe stenosis of both ICA and the A1 segments. Also, moderate stenosis of the left MCA was obvious with pronounced collaterals between the ICAs, both proximal ACAs and the right posterior communicating artery (PCOM). The dilated perforating vessels in the basal ganglia and hypothalamus were supposed to be caused by hypoperfusion due to MMD. One distinctive large collateral vessel was present between the reticular collaterals of the distal right-sided ICA and the ACA territory bearing a small aneurysm (Fig. 2). Given the primary blood distribution, this 2 mm-sized wide neck aneurysm was suspected as bleeding source. Thus, direct occlusion of the deep-seated aneurysm by means of either surgical or endovascular was considered to be associated with very high treatment-related morbidity, so that revascularization of the right hemisphere was indicated as the first step in order to reduce collateral perfusion in the perforating arteries. Due to a potentially increased perioperative risk in the acute phase after SAH and IVH, bypass surgery was scheduled 4 weeks later when the ICH had completely resorbed and the patient clinically recovered.

Blood pressure was monitored and systolic peak values above 140 mmHg were prevented medically to avoid rebleeding. Nimodipine (dosage six times 60 mg per day) was administered as a precaution against vasospasm/delayed cerebral ischemia and daily transcranial Doppler ultrasound revealed no signs of vasospasm. After 4 weeks, low-dose aspirin was started and standard extracranial-intracranial (EC-IC) bypass surgery with a supplementary encephalomyosynangiosis was performed via a right-sided frontotemporal surgical approach. The patient rapidly recovered from surgery but complained about severe headache and nausea. Postoperative CT and CTA revealed a small rehemorrhage in the right hypothalamus again with intraventricular extension and a patent anastomosis. Additionally, a dissection of the left vertebral artery (VA) was discovered in the V3-segment and was treated with unfractionated heparin as standard of care. The underlying cause for the vessel dissection remained unclear. The following week, the patient was clinically stable without signs of vasospasm, ischemia, or hydrocephalus.

**Fig. 3:**
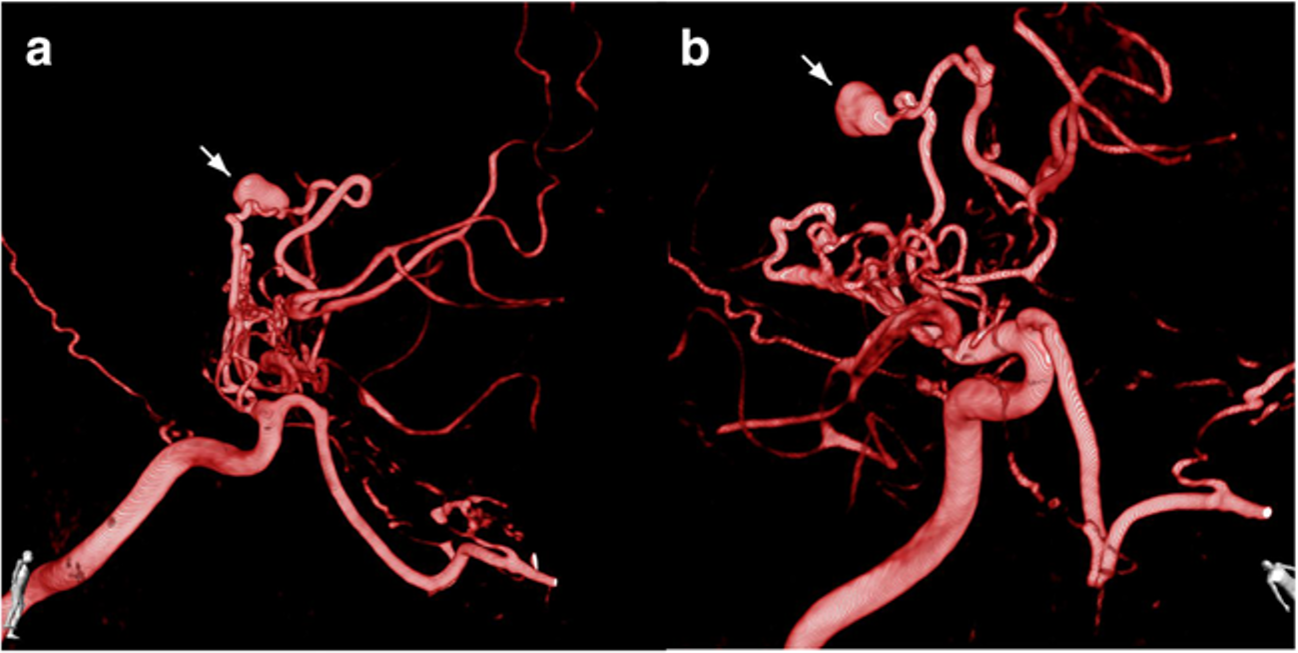
The 3D reconstruction of the follow-up CT angiogram showing the configuration of the collateral vessel aneurysm

Postoperative DSA 1 week after surgery demonstrated proper bypass function with filling of the MCA territory and reduced perfusion of the network of collaterals around the ICA and proximal right-sided ACA. The aneurysm of the fragile collateral vessel, however, grew significantly within the following 3 weeks to a size of 7 mm (Fig. 3). In view of the rapid aneurysm growth and repeated hemorrhage, the rebleeding risk was assumed high and treatment was considered mandatory. Given the limited endovascular options with potential occlusion of the perforator and consecutive ACA ischemia, surgical exploration was indicated.

**Fig. 4:**
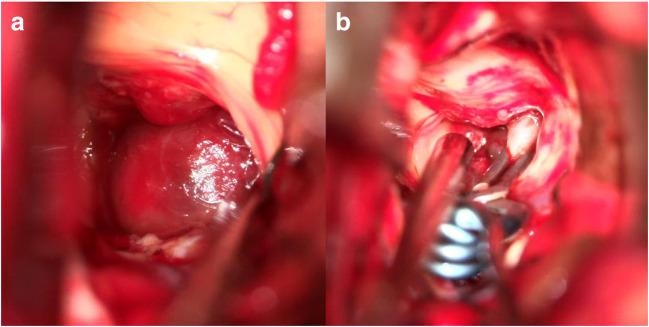
Intraoperative pictures via the third ventricular approach shows the aneurysm **a** in situ and **b** after microsurgical clipping

**Fig. 5:**
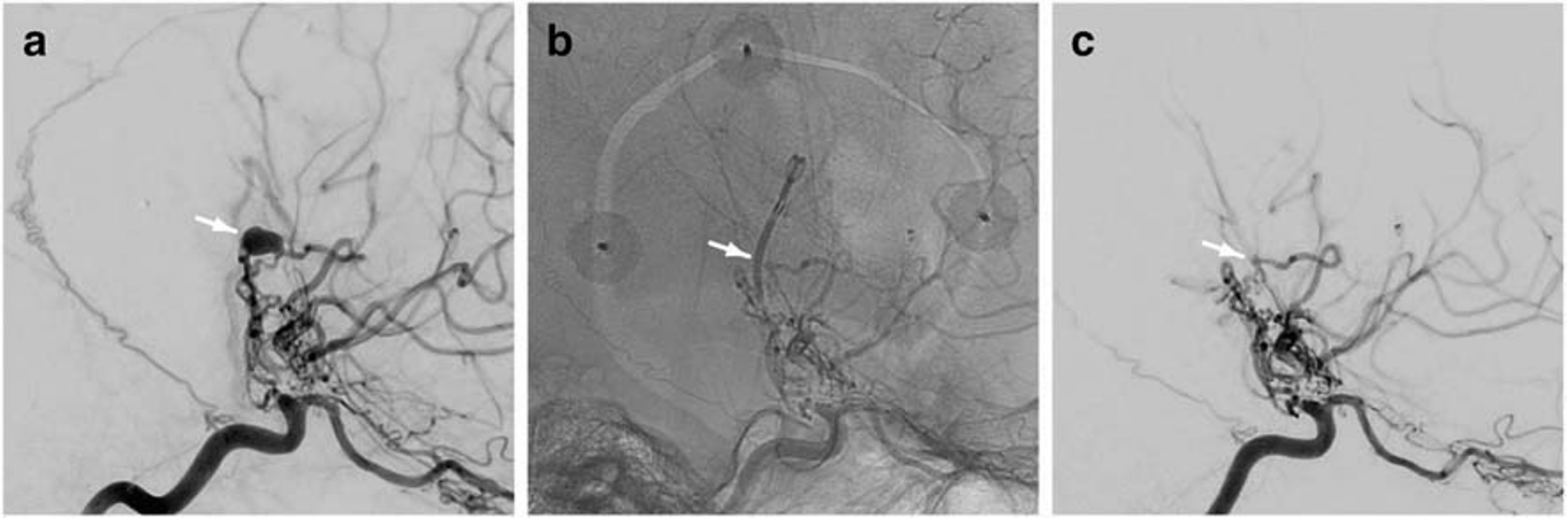
The arrow in the DSA points to **a** the aneurysm before surgery, **b** the aneurysm clip, and **c** the complete obliteration of the aneurysm

One month after EC-IC bypass surgery, the aneurysm was exposed using neuronavigation via an interhemispheric transcallosal, right-sided transchoroidal approach through the third ventricle. The aneurysm was surrounded by residual intraparenchymal hemorrhage and mobilized out of the hypothalamus, so that direct clipping could be achieved (Fig. 4). DSA confirmed complete aneurysm occlusion while showing the parent vessel intact (Fig. 5). The VA dissection was still stenotic but sufficiently collateralized via the contralateral side. There were no signs of hemorrhage or ischemia with no new symptoms or neurologic deficits postoperatively. The patient was discharged home neurologically intact and in a very good overall clinical condition.

## Discussion

To the best of our knowledge, this is the first report of a direct clipping of a perforator aneurysm in the hypothalamus via an interhemispheric transcallosal approach. Of note, this approach has been described in the treatment of both arteriovenous and cavernous malformations [3, 5, 6].

MMD-associated collateral artery aneurysms are usually treated indirectly by surgical revascularization causing aneurysm obliteration due to an alteration of flow-related vessel dynamics [4, 11, 14, 15, 17, 20]. The timing of bypass surgery after the initial bleeding remains unclear. Kanamori et al. recommend the operation as soon as the patients are stable, since collateral artery aneurysms localized around the ventricular walls in MMD are prone to rerupture within 1 month [11]. In our case, we accordingly performed an EC-IC bypass, yet the aneurysm size significantly increased within a short period of time. Cases of aneurysm formation localized close to the anastomosis after bypass surgery have been documented [1, 7, 8, 16, 19], but to date, only few cases of aneurysm formation and progression in collateral MMD vessels have been reported [10, 12, 21].

The treatment of deep-seated peripheral artery aneurysms is technically challenging [22]. Either the feeding artery is hardly accessible for endovascular coiling due to the small size of the vessel, the fusiform character of the lesion and the fragile vascular structure, or the surgical approach may entail parenchymal damage of eloquent brain areas. Both treatment modalities bear significant risks of procedure-related adverse events. Only few cases of deep-seated peripheral artery aneurysms have been reported with favorable outcomes [9]. In general, surgery within the hypothalamus is associated with a high risk of perioperative and postoperative morbidity including endocrinological and even neuropsychological deficits. In our case, the patient underwent direct aneurysm clipping without surgery-related complications and recovered completely without any neurological deficits. This may be due to the repeated intraparenchymal hemorrhage with rupture into the third ventricle, which has created an intraoperatively visible rather safe entry zone into the hypothalamus and may have eased the mobilization of the aneurysm into the third ventricle. Although proximal control could not be obtained without entering the hypothalamus further, intraoperative rupture was easily controlled, as flow in the perforators is somewhat limited. Therefore, surgical exploration is a feasible and safe option in cases in which fail revascularizations show repeated hemorrhage and/or are not amenable to endovascular treatment.

## Conclusion

In cases of deep-seated peripheral collateral artery aneurysms localized in the hypothalamus, microsurgical clipping via a navigated interhemispheric transcallosal, transchoroidal approach through the third ventricle is technically feasible with favorable neurological outcome.

## Electronic supplementary material

Supplementary Fig. 1 T1-weighted MRI with contrast showing the aneurysm located in the hypothalamus in **a** axial, **b** the Moya-Moya vessels, **c** coronar, and **d** sagittal view (JPEG 90 kb)

Supplementary Fig. 2 T2-weighted MRI presenting **a** signs of leukoencephalopathy, **b** the ruptured aneurysm, **c** the Moyamoya vessels (JPEG 97 kb)
