# Supplementary figures and images for: Transcallosal, transchoroidal clipping of a hypothalamic collateral vessel aneurysm in Moyamoya disease
Source: Acta Neurochir (Wien). 2020 Apr 19;162(8):1861–5. doi: 10.1007/s00701-020-04335-4 (PMC7360665; doi:10.1007/s00701-020-04335-4)

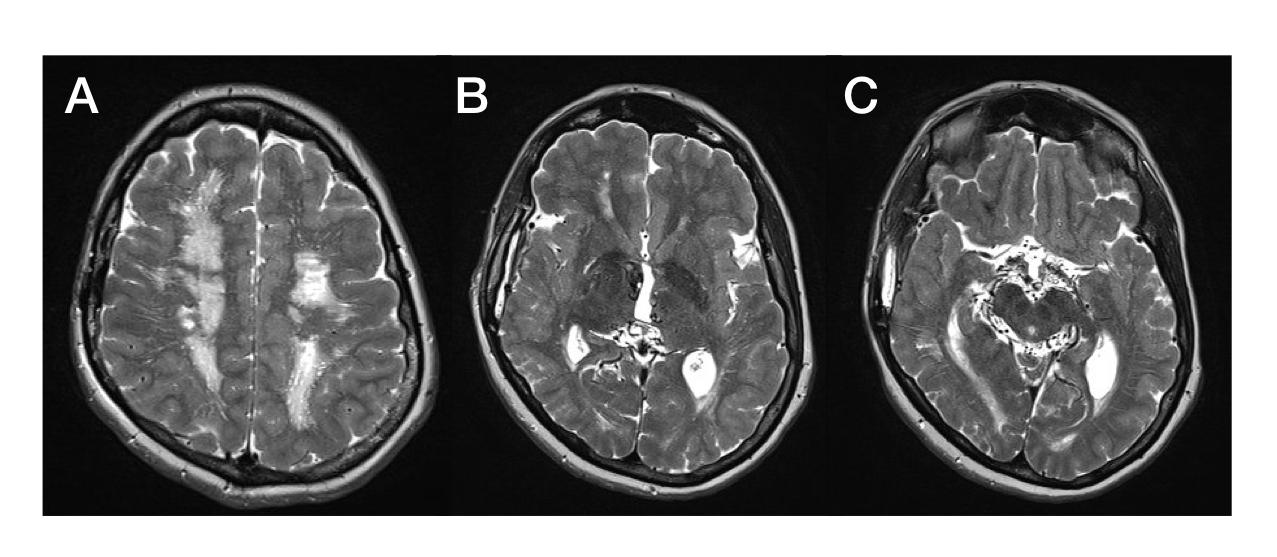

Supplement: Supplementary file 1 — T1-weighted MRI with contrast showing the aneurysm located in the hypothalamus in a axial, b the Moya-Moya vessels, c coronar, and d sagittal view (JPEG 90 kb) [file 701_2020_4335_MOESM1_ESM.jpg]

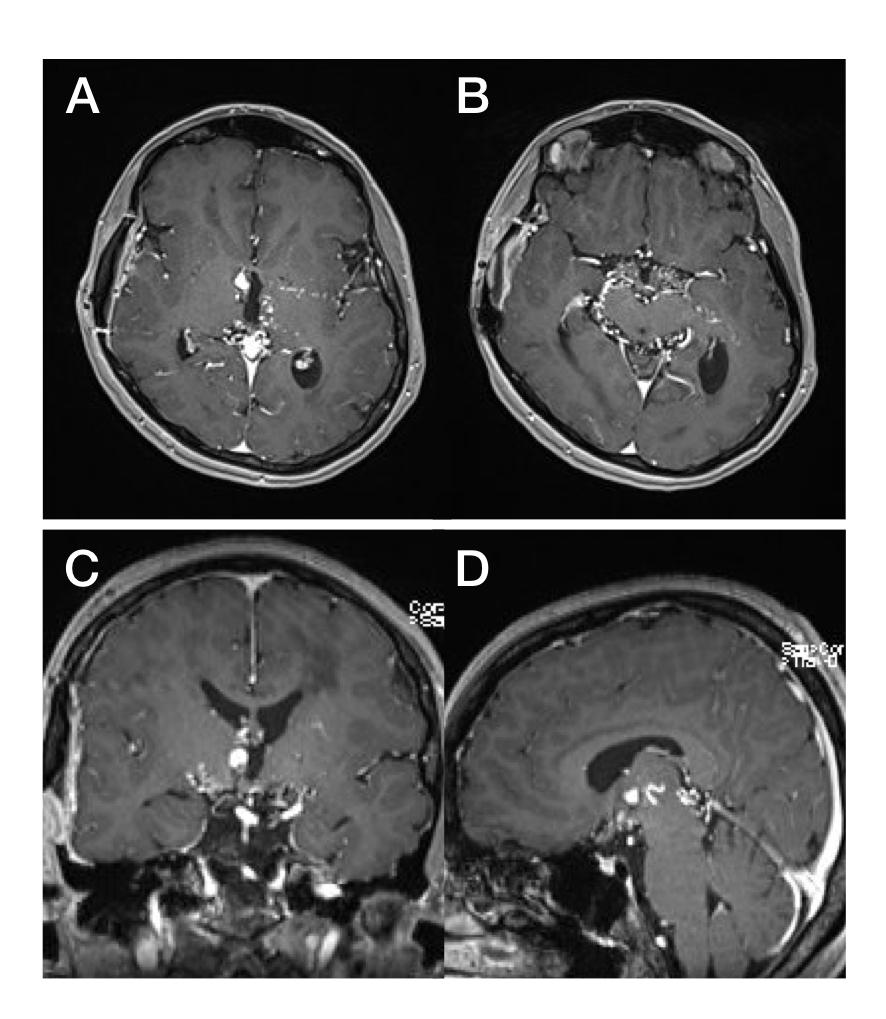

Supplement: Supplementary file 2 — T2-weighted MRI presenting a signs of leukoencephalopathy, b the ruptured aneurysm, c the Moyamoya vessels (JPEG 97 kb) [file 701_2020_4335_MOESM2_ESM.jpg]
